# Recruiting health professionals to the COVID-19 response, Brazil

**DOI:** 10.2471/BLT.22.288060

**Published:** 2022-06-13

**Authors:** Ianka Cristina Celuppi, Geovana dos Santos Lima, Alessandra Castro, Joao Pedro T Souza, Leonardo Mello, Gustavo Hoff, Mariano Felisberto, Celio Luiz Cunha, Jades Fernando Hammes, Raul Sidnei Wazlawick, Eduardo M Dalmarco

**Affiliations:** a Bridge Laboratory, Federal University of Santa Catarina, Lauro Linhares Street, Florianópolis, 88040-370, Brazil.; b Department of Health Work Management, Ministry of Health, Brasilia, Brazil.

## Abstract

**Problem:**

The coronavirus disease 2019 (COVID-19) pandemic posed a major workforce challenge to Brazil, which has a large land area and a shortage of health workers in regions distant from the big cities.

**Approach:**

The Brazilian health ministry implemented a computerized solution to provide rapid support to states and municipalities to hire health professionals from large urban centres to work in underserved areas during the COVID-19 pandemic. We designed an online system for health professionals to register their willingness to work on the COVID-19 response; the system was launched to the public in April 2020.

**Local setting:**

Brazil is a large country with great heterogeneity in access to health care across its different regions. Before the initiative was launched, 5 156 020 health professionals were officially registered with professional councils. However, an estimated 3 200 000, more than 60% of them, were working in the two regions with the highest standard of living.

**Relevant changes:**

Up to February 2022, 1 007 138 health professionals had self-registered on the system, providing a sizeable database of professionals from a range of disciplines. Of these, 371 275 professionals were willing to work on the COVID-19 response in remote areas. By 1 February 2022, 157 755 professionals have been trained and deployed to these underserved areas.

**Lessons learnt:**

Partnership of the government with professional councils and the use of official communication channels were important strategies to improve registration and ensure the success of the scheme. We predict that the database will assist with future public health campaigns in Brazil.

## Résumé

**Problème:**

La pandémie de maladie à coronavirus 2019 (COVID-19) a constitué un enjeu majeur pour le Brésil en termes de main-d'œuvre. En effet, le pays couvre un vaste territoire et manque de travailleurs de la santé dans les régions éloignées des grandes métropoles.

**Approche:**

Le ministère de la Santé brésilien a développé une solution informatique destinée à fournir une assistance rapide aux États et municipalités ayant besoin d'engager des professionnels de la santé issus des principaux centres urbains, afin qu'ils exercent dans les zones mal desservies durant la pandémie de COVID-19. Nous avons conçu un système en ligne pour ces professionnels, leur permettant de manifester leur intérêt pour la lutte contre la COVID-19; le système est entré en service en avril 2020.

**Environnement local:**

Le Brésil est un immense pays, où l'accès aux soins de santé varie énormément d'une région à l'autre. Avant le lancement de l'initiative, 5 156 020 travailleurs de la santé étaient officiellement enregistrés auprès des conseils représentant leur profession. Cependant, on estime que 3 200 000 d'entre eux, c'est-à-dire plus de 60%, pratiquaient leur métier dans les deux régions offrant le meilleur niveau de vie du pays.

**Changements significatifs:**

En février 2022, 1 007 138 professionnels de la santé s'étaient inscrits dans le système, générant une base de données considérable de spécialistes de différents domaines. Parmi eux, 371 275 étaient prêts à s'engager dans la lutte contre la COVID-19 dans les zones reculées. Au 1^er^ février 2022, 157 755 professionnels avaient été formés et déployés dans ces régions mal desservies.

**Leçons tirées:**

La collaboration entre le gouvernement et les conseils professionnels, ainsi que l'utilisation des canaux de communication officiels, sont autant de stratégies essentielles pour améliorer l'enregistrement et garantir le succès du programme. Selon nos prévisions, la base de données se révélera utile à l'organisation de futures campagnes de santé publique au Brésil.

## Resumen

**Situación:**

La pandemia de la enfermedad por coronavirus de 2019 (COVID-19) supuso un importante reto de personal para Brasil, que tiene una gran extensión territorial y una escasez de personal sanitario en las regiones alejadas de las grandes ciudades.

**Enfoque:**

El Ministerio de Salud de Brasil implementó una solución informática para proporcionar apoyo rápido a los estados y municipios a fin de contratar a profesionales sanitarios de los grandes centros urbanos para trabajar en las áreas desatendidas durante la pandemia de la COVID-19. Así, se diseñó un sistema virtual para que los profesionales sanitarios registraran su disposición a trabajar durante la respuesta a la COVID-19; el sistema se puso a disposición del público en abril de 2020.

**Marco regional:**

Brasil es un país extenso con una gran heterogeneidad en el acceso a la asistencia sanitaria en sus diferentes regiones. Antes de la puesta en marcha de la iniciativa, 5 156 020 profesionales sanitarios estaban registrados oficialmente en los consejos profesionales. No obstante, se calcula que 3 200 000, más del 60 % de ellos, trabajaban en las dos regiones con mayor nivel de vida.

**Cambios importantes:**

Hasta febrero de 2022, 1 007 138 profesionales sanitarios se habían inscrito en el sistema, lo que supone una importante base de datos de profesionales de diversas disciplinas. De ellos, 371 275 profesionales estaban dispuestos a trabajar durante la respuesta a la COVID-19 en las áreas remotas. A 1 de febrero de 2022, 157 755 profesionales habían sido formados y desplegados en estas áreas desatendidas.

**Lecciones aprendidas:**

La asociación del gobierno con los consejos profesionales y el uso de los canales de comunicación oficiales fueron estrategias importantes para mejorar el registro y asegurar el éxito del sistema. Se prevé que la base de datos ayude en futuras campañas de salud pública en Brasil.

## ملخص

المشكلة كانت جائحة مرض فيروس كورونا 2019 (كوفيد 19) تمثل تحديًا رئيسيًا للقوى العاملة للبرازيل، والتي لديها مساحة كبيرة من الأرض ونقص في العاملين في المجال الصحي في المناطق البعيدة عن المدن الكبرى.

الأسلوب قامت وزارة الصحة البرازيلية بتنفيذ حل محوّسب لتوفير الدعم السريع للولايات والبلديات، بهدف توظيف أخصائيين للصحة من المراكز الحضرية الكبيرة للعمل في المناطق المحرومة أثناء جائحة كوفيد 19. لقد وضعنا نظامًا عبر الإنترنت لأخصائيي الصحة لتسجيل رغبتهم في العمل في مواجهة كوفيد 19. وتم إطلاق النظام للجمهور في أبريل/نيسان 2020.

المواقع المحلية البرازيل دولة كبيرة تشهد حالة من عدم التجانس الهائل في الحصول على الرعاية الصحية عبر مناطقها المختلفة. قبل إطلاق المبادرة، تم تسجيل 5156020 أخصائيًا صحيًا بشكل رسمي في المجالس المهنية. إلا أن قرابة 3200000، أي أكثر من 60% منهم، كانوا يعملون في المنطقتين اللتين تتمتعان بأعلى مستوى للمعيشة.

التغيّرات ذات الصلة وصولًا إلى فبراير/شباط 2022، قام 1007138 أخصائيًا صحيًا بالتسجيل الذاتي لأنفسهم في النظام، مما وفر قاعدة بيانات ضخمة للأخصائيين من مجموعة من التخصصات. ومن بين هؤلاء، كان 371275 أخصائيًا يرغبون في العمل على مواجهة كوفيد 19 في المناطق النائية. بحلول 1 فبراير/شباط 2022، تم تدريب 157755 أخصائيًا، وتوزيعهم على هذه المناطق المحرومة.

الدروس المستفادة إن الشراكة بين الحكومة والمجالس المهنية، واستخدام قنوات الاتصال الرسمية، كانت من الاستراتيجيات الهامة لتحسين التسجيل وضمان نجاح الخطة. نحن نتوقع أن تساعد قاعدة البيانات في حملات الصحة العامة المستقبلية في البرازيل.

## 摘要

**问题:**

新型冠状病毒病肺炎 (COVID-19) 大流行给巴西劳动力市场带来巨大挑战，由于巴西幅员辽阔，许多远离大城市的地区都缺少卫生工作者。

**方法:**

巴西卫生部实施了一种计算机化的解决方案，可以为各州和市政当局提供快速支持，以供新冠肺炎大流行期间从大型城市中心雇用卫生专业人员在医疗服务匮乏的地区工作。我们为卫生专业人员设计了一个在线系统，用于登记他们参与新冠肺炎疫情应对工作的意愿；系统于 2020 年 4 月向公众推出。

**当地状况:**

巴西是一个大国，各个地区的医疗覆盖率存在很大差异。倡议发起前，已有 5,156,020 名卫生专业人员在专业委员会正式登记。然而，估计其中有 3,200,000 名（超过 60% ）卫生人员在生活水平最高的两个地区工作。

**有关变化:**

截至 2022 年 2 月，有 1,007,138 名卫生专业人员已在系统上自主注册，形成了由各个学科专业人员组成的庞大数据库。其中 371,275 名专业人员愿意前往偏远地区从事新冠肺炎疫情应对工作。截至 2022 年 2 月 1 日，157,755 名专业人员已接受培训并被分配到这些医疗服务匮乏的地区。

**经验教训:**

政府与专业委员会合作以及使用官方沟通渠道是提高注册率和确保计划成功的重要策略。我们预测该数据库将有助于巴西未来的公共卫生活动。

## Резюме

**Проблема:**

Пандемия коронавирусной инфекции 2019 года (COVID-19) создала серьезную проблему с трудовыми ресурсами для Бразилии, которая имеет большую территорию и испытывает нехватку работников здравоохранения в регионах, удаленных от крупных городов.

**Подход:**

Министерство здравоохранения Бразилии внедрило компьютеризированное решение для оказания оперативной поддержки штатам и муниципалитетам в вопросе найма медицинских работников из крупных городских центров для работы в районах, не получающих надлежащего медицинского обслуживания во время пандемии COVID-19. Авторы разработали онлайн-систему для медицинских работников, чтобы они могли регистрировать свою готовность участвовать в борьбе с COVID-19. Система была представлена общественности в апреле 2020 года.

**Местные условия:**

Бразилия — большая страна с неодинаковым доступом к медико-санитарной помощи в разных регионах. До запуска инициативы 5 156 020 медицинских работников были официально зарегистрированы в профессиональных советах. Однако, по оценкам, 3 200 000 человек (более 60% из них) работали в двух регионах с самым высоким уровнем жизни.

**Осуществленные перемены:**

По состоянию на февраль 2022 года 1 007 138 медицинских работников самостоятельно зарегистрировались в системе, что позволило создать обширную базу данных специалистов из различных областей. Из них 371 275 специалистов были готовы участвовать в борьбе с COVID-19 в отдаленных районах. К 1 февраля 2022 года 157 755 специалистов прошли обучение и были направлены в районы, не получающие надлежащего медицинского обслуживания.

**Выводы:**

Сотрудничество правительства с профессиональными советами и использование официальных каналов связи были важными стратегиями улучшения регистрации и обеспечения успеха схемы. Авторы прогнозируют, что эта база данных поможет в будущих кампаниях в области общественного здравоохранения в Бразилии.

## Introduction

The coronavirus disease 2019 (COVID-19) outbreak was declared a pandemic by the World Health Organization in early 2020. Two years on, the pandemic still represents a major challenge for health systems, which must implement strategies to combat the spread of infection, restructure the health service network to meet the increased demand for treatment of respiratory symptoms, and continue to offer care for other health conditions.^1^ Brazil too faced challenges such as a high demand for care, lack of hospital beds, illness of professionals, long-standing shortages of human resources and materials, and the need for professional training on the clinical management of people infected with COVID-19.^2^^,^^3^

We describe how the Brazilian government implemented a computerized strategy to link health councils, health professionals and the government in the selection and assignment of health professionals to the most isolated and underserved regions of the country.

## Local setting

The total population in Brazil is around 213.48 million people. Brazil’s unified health system was established by the Brazilian Federal Constitution in 1988.^4^ Since then, Brazil’s government has decentralized tasks and resources and increased the supply and access to health services, which has had a positive impact on the population’s health and mortality.^5^^,^^6^ However, public health managers face some challenges in the implementation of the health policy, such as a lack of public infrastructure, lack of planning, difficulties in equitable distribution of resources across regions, lack of human resources in some areas, and resistance to changes in health-care models and practices.^5^ While the two more developed regions of Brazil in terms of human development index have an estimated 3 200 000 (62%) of the 5 156 020 total health workforce,  the two least developed regions have no more than about 700 000 (14%) of these professionals. 

To meet the challenge of maintaining health assistance to all regions of the country during the COVID-19 pandemic, the health ministry coordinated workforce management policies and provided health professionals and institutional support to states and municipalities of the country. For this, the health ministry designed the initiative *O Brasil Conta Comigo*^7^ to provide institutional support for the recruitment of health professionals during the COVID-19 pandemic.

## Approach

The initiative was instituted through a government ordinance on 31 March 2020,^7^ with the objective of training health professionals on COVID-19 clinical management protocols, after which they could participate in the national COVID-19 programme. 

We designed an online system that would allow health professionals to self-register to work on the COVID-19 response. The aim was to create a database to support decisions on hiring health professionals by local health authority managers, thus facilitating the recruitment process, and ensuring that professionals were trained on clinical management protocols for COVID-19 infections. We developed the registration system following the model–view–controller architecture pattern, with Spring Framework (VMware, Palo Alto, United States of America), Java 8 (Oracle Corporation, Santa Clara, USA) and React (Facebook open source Meta platforms, Menlo Park,  USA) languages. We chose the Postgres (PostgreSQL Global Development Group, Berkeley, USA) database management system because it is open-source and simple to use.

All health professionals registered with their respective health councils were eligible to participate in the strategy, regardless of whether they were public or private employees. If the professional was already in work, the central government was allowed (if the employee agreed) to request that the employer release the employee to work on the COVID-19 response. The system accepted the registration of the following professional categories to work in their area of expertise if their professional registration was up to date: biology, biomedicine, physical education, nursing, pharmacy, physiotherapy, occupational therapy, speech therapy, medicine, veterinary, nutrition, dentistry, psychology and radiology. To implement the initiative, it was necessary to rely on the assistance of professional councils, who provided the health ministry with a list of professionals to enable a comparative analysis and validation of registration information.

The information collected during registration and stored in the database can be divided into three sections: personal, professional and data relating to actions to combat COVID-19. The first section focuses on the storage of identifying and contact information. The second section focuses on data about the employment relationship, which facilitates analysing the professional’s profile and for orienting and structuring future health ministry actions. The third section focuses on data collected about the professionals assigned to initiatives related to COVID-19. This section helps in the mapping of some conditions, such as the number of professionals with suspected or confirmed COVID-19 infection and the number of professionals working directly in the field. The entire registration process is done on a freely accessible, public website under the health ministry domain.

The database includes a dashboard which is used exclusively by the health ministry and allows data to be extracted on professionals who completed registration, those willing to act in initiatives to combat COVID-19, those willing to act in other initiatives, those who are not directly dealing with suspected or confirmed cases of COVID-19, and those who have completed training. It is possible to analyse the records by professional category, view the total number of records and their relationship with the total number of professionals registered by state or region in the regional councils, and present a percentage of records by category. It is also possible to obtain the recruitment status for each action registered in the system and analyse the number of professionals that the initiative needs and the numbers already recruited, remaining to be recruited, with a signed contract, being hired, on leave and with contracts finished.

After the launch of the registration tool in April 2020, the health ministry, in partnership with the professional councils, publicized the initiative and encouraged adherence to registration requirements through internal and external means of communication, such as social media and media channels. 

In February 2022 we performed an analysis of the numbers of professionals registered and willing to work on the COVID-19 response by region and state. The health ministry maintains restricted and controlled access to the information. The data were shared with us, as developers of the registration system upon authorization of use for scientific purposes, thus respecting the principles of confidentiality and anonymity of the data. 

## Relevant changes

The tool for the registration of professionals was made available to the public on 2 April 2020. Within 6 months, the database grew to more than 1 million subscribers. Of these, more than 346 000 subscribers received training on the protocol for clinical management of COVID-19. By analysing the health ministry’s database, we could identify the professionals, their specializations, place of residence and availability to participate in the COVID-19 care programme.

Up to February 2022, the database had 1 007 566 health professionals registered out of the total 5 156 020 health professionals registered with health councils. Table 1 shows the total number of professionals registered by professional category and the percentage of registrations out of the total number of health professionals registered in each health council in the country.

**Table 1:** Total number of registrations in the initiative to recruit health professionals to the COVID-19 response, Brazil, February 2022

Professional council	Total no. of health professionals	No. (%) fully registered in the initiative
Federal Council of Nursing	2 262 846	159 735 (7.1)
Federal Council of Physiotherapy and Occupational Therapy	155 390	134 911 (86.8)
Federal Council of Dentistry	329 432	133 742 (40.6)
Federal Council of Pharmacy	331 007	113 001 (34.1)
Federal Council of Nutrition	149 025	98 691 (66.2)
Federal Council of Physical Education	450 000	83 561 (18.6)
Federal Council of Veterinary	139 596	69 966 (50.1)
Federal Council of Psychology	363 847	64 518 (17.7)
Federal Council of Medicine	496 997	35 216 (7.1)
Federal Council of Biomedicine	57 665	28 697 (49.8)
Federal Council of Speech Therapy	45 123	24 810 (55.0)
Federal Council of Social Work	190 693	22 933 (12.0)
Federal Council of Biology	63 095	21 962 (34.8)
National Council of Radiology Technicians	121 304	15 813 (13.0)
**Total**	**5 156 020**	**1 007 566 (19.5)**

COVID-19: coronavirus disease 2019.

Source: Data from the professional councils.

The total number of registrations by region and state in Brazil are represented in Table 2. The south-east region had the highest number of registrations of health professionals (498 345 people, 49.4%) followed by the south (187 293 people, 18.5%), north-east (185 818 people, 18.4%), mid-west (83 639 people, 8.3%) and north (52 471 people, 5.2%). The south and south-east regions together have a higher human development index compared with other regions of the country.^8^

**Table 2:** Health professionals registered and willing to work on the COVID-19 response in remote areas by states and regions in Brazil, February 2022

Estate or State	Population^a^	No. of health professionals	
		Completed registration	Willing to work on the COVID-19 response in remote areas
**North region**			
Acre	733 559	2 444	1 440
Amapá	669 526	3 898	1 990
Amazonas	3 483 985	15 951	8 559
Pará	7 581 051	16 843	8 162
Rondônia	1 562 409	6 351	3 083
Roraima	450 479	2 261	1 115
Tocantins	1 383 445	4 723	2 084
**North-east region**			
Alagoas	3 120 494	10 841	4 887
Bahia	14 016 906	48 644	20 515
Ceará	8 452 381	28 999	12 207
Maranhão	6 574 789	12 842	6 002
Paraíba	3 766 528	17 802	6 620
Pernambuco	8 796 448	32 351	12 518
Piauí	3 118 360	11 067	4 472
Rio Grande do Norte	3 168 027	13 980	6 245
Sergipe	2 068 017	9 292	3 903
**Mid-west region **			
Federal District	2 570 160	27 756	13 573
Goiás	6 003 788	28 528	11 385
Mato Grosso	3 035 122	13 360	5 730
Mato Grosso do Sul	2 449 024	13 995	5 840
**South-east region**			
Espírito Santo	3 514 952	20 686	7 453
Minas Gerais	19 597 330	99 753	35 666
Rio de Janeiro	15 989 929	91 472	31 215
São Paulo	41 262 199	286 434	92 993
**South region**			
Paraná	8 912 692	62 035	21 404
Rio Grande do Sul	10 693 929	76 510	26 556
Santa Catarina	6 248 436	48 748	15 628
**Total**	**189 223 965**	**1 007 566**	**371 275**

COVID-19: coronavirus disease 2019.

^a^ Based on the last official census in 2010.

Source: Data from the health ministry dashboard extracted on 16 February 2022.

Of the total registrations, 371 275 professionals were willing to work on the COVID-19 response in remote areas. By 1 February 2022, approximately 157 755 health professionals have been deployed to underserved areas to care for patients with COVID-19. 

## Lessons learnt

With the shortage of human resources in health services during the COVID-19 pandemic, this initiative supported the recruitment of professionals and expanded the supply of care at a time of great need. In addition, the initiative contributed and continues to contribute to the training of thousands of professionals to assist in the clinical management of COVID-19 in Brazil, thus improving the health care provided to the population. The results demonstrate the positive impact of the strategy in making human resources available to the northern region of Brazil, where the health system was severely affected by the high demand for the treatment of respiratory symptoms and high mortality rates.^9^ The health workers will remain in underserved areas for as long as they are needed or as long as they wish to continue.

The low participation of health professionals in the online registration was an initial obstacle we encountered during implementation of the initiative. In an attempt to mitigate this problem, the health ministry chose to adopt a strategy of wide dissemination of the initiative, partnerships with professional councils and the use of official communication channels in addition to print, television and digital media (Box 1). Another obstacle was difficulty in accessing the internet for staff in some regions of Brazil. It is likely that not all health professionals were familiar with digital media, which may have influenced the total number of registrations.

Box 1 **Summary of main lessons learnt**

- Constructing a database of health-care professionals helped in the recruitment and distribution of human resources for the COVID-19 response in a middle-income country with a large land area.
- Small municipalities with few resources or situated far from urban centres took advantage of the online registration system as a tool for public health management decision-making during the public health emergency.
- The partnership established with professional health councils for publicizing the registration system enhanced the government’s initiative.

COVID-19: coronavirus disease 2019.

The development of the registration tool took place in just one month. Therefore, it was necessary to implement improvements after the tool was launched, and with it the inclusion of new registration fields. To solve this problem, emails were sent to registered professionals requesting them to update their data on the system.

The initiative focused attention on usability and ease of registration by health professionals without jeopardizing the security of the data entered in the system. We predict that the database will assist with other public health needs in Brazil. Possible uses include the recruitment of professionals for vaccination campaigns, actions aimed at riverside populations and task-force groups to act in situations of natural disasters.
